# Unravelling the Complexity of Biofilms—New Mechanistic Insights and Control Strategies

**DOI:** 10.3390/ijms262211088

**Published:** 2025-11-16

**Authors:** Lúcia Chaves Simões, Manuel Simões

**Affiliations:** 1 Department of Biology, University of Minho, Campus de Gualtar, 4710-057 Braga, Portugal; 2 LEPABE—Laboratory for Process Engineering, Environment, Biotechnology and Energy, ALiCE—Associate Laboratory in Chemical Engineering, Faculty of Engineering, University of Porto, Rua Dr. Roberto Frias, 4200-465 Porto, Portugal; mvs@fe.up.pt

The advances in biofilm research have provided the scientific community with a clear understanding that biofilm formation is not simply a matter of cells sticking to surfaces but rather a dynamic, regulated process involving adhesion, cell communication, the production of extracellular polymeric substances, community maturation, spatial heterogeneity, dispersal, and interactions with the environment and host. Biofilms are being increasingly recognized as dynamic, structured communities that challenge conventional antimicrobial strategies and pose significant challenges across health, industrial, environmental, and agricultural sectors [1,2]. Over the past few decades, remarkable progress in biofilm research has transformed our understanding of these complex systems. Advances in molecular tools, microscopy, omics technologies, and modeling have enabled researchers to unravel the intricacies of the biofilm lifecycle—from initial adhesion and cell signaling to matrix production, cellular heterogeneity, dispersal, persistence, and recalcitrance [3,4]. However, an effective strategy for biofilm control—causing activation and removal—remains to be identified. The second edition of the Special Issue: Mechanisms in Biofilm Formation, Tolerance, and Control seeks to highlight cutting-edge research that deepens our understanding of three key areas: how biofilms form, how they tolerate and resist treatments, and how we can most effectively disrupt or control them.

This Special Issue is composed of one opinion article, two review articles, and seven original research articles, expanding our current knowledge on biofilm formation, structure, virulence, control, and tolerance. Below we provide a brief statement of each manuscript included in this Special Issue.

An opinion/perspective article examines how immune cell–biofilm interactions reshape our definition of a biofilm, arguing for deeper integration of immunological context into biofilm research to achieve more realistic mechanistic models and effective therapies (contribution 1). An extensive review synthesizes the role of outer-membrane vesicles (OMVs) in biofilm formation, virulence, and antimicrobial resistance in the critical ESKAPE pathogen group. It offers a relevant mechanistic framework for the interplay between OMVs and biofilm and identifies therapeutic gaps in this emerging research area (contribution 2). Tegegne et al. (contribution 3) provided a consolidated knowledge base on catheter-associated urinary tract infections, biofilm formation on indwelling devices, antimicrobial resistance mechanisms, and emerging in vitro models. This authoritative review emphasizes the translation of mechanistic insights into clinical prevention and therapy. 

In terms of original research, Liu et al. (contribution 4) dissected the distinct roles of the Psl, Pel, and alginate EPS pathways in *Pseudomonas aeruginosa* biofilm architecture, showing how each contributes to biofilm thickness, mechanical stability, and antimicrobial tolerance, thereby refining our mechanistic understanding of EPS regulation in a major opportunistic pathogen. Poli et al. (contribution 5) uncover the pivotal role of the c-di-GMP effector protein BpfD in regulating pellicle biosynthesis in *Shewanella oneidensis*, offering new molecular insights into biofilm formation and its control mechanisms in this environmentally and biotechnologically significant bacterium. A genome-wide analysis of *S. aureus* isolates from chronic rhinosinusitis patients identified core and accessory virulence gene clusters linked to biofilm-associated persistence and immune evasion, offering mechanistic insights into strain variability and biofilm-driven disease (contribution 6). Through in vitro and in vivo experiments, Phuengmaung et al. (contribution 7) revealed that mixed-species biofilms of *Klebsiella pneumoniae* and *Candida albicans* produce more biomass but paradoxically trigger lower neutrophil activation and lung inflammation compared to *K. pneumoniae* alone, highlighting the complexity of host–biofilm interactions and the need to consider polymicrobial biofilm behavior. Mechmechani et al. (contribution 8) compared the resistance of *Staphylococcus aureus* biofilms, detached biofilm cells, and planktonic cells to microencapsulated carvacrol, either alone or in combination with low-pH treatment. They found that while biofilms are more resilient, the combinatorial treatment achieved a reduction of more than 5 log CFU/mL, demonstrating a potential method for biofilm eradication in sensitive settings. Further innovation in biofilm control was reported, where halogenated pyrimidine derivatives were screened for antibiofilm efficacy, demonstrating significant downregulation of curli genes and motility (key factors in enterohemorrhagic *Escherichia coli* biofilm formation) without inhibiting planktonic growth—underscoring a targeted strategy for biofilm control in food safety contexts (contribution 9). Investigating a plant-associated bacterium, Rajewska et al. (contribution 10) demonstrated that the carbon source (e.g., glycerol vs. glucose) and the nature of the substratum significantly influence biofilm formation and architecture. These findings highlight that laboratory surface assays may not directly translate to environmental or host colonization contexts. Collectively, the manuscripts in this Special Issue advance current knowledge in biofilm science and technology, ranging from the molecular intricacies of pathways involved in the synthesis of extracellular polymeric substances to the pragmatic challenges of device-associated biofilms, from single-species models to polymicrobial communities. Crucially, they reflect the multidisciplinary approach that modern biofilm science demands, incorporating microbiology, materials and surface science, immunology, chemistry, and engineering. We hope this collection serves as both a reference point for current understanding and encouragement for future innovation in tackling biofilms. We extend our gratitude to all authors, reviewers, and the MDPI team for their contributions to this volume. We invite you to engage with each of these articles, to build on their insights, and to drive forward advances in biofilm research.

## References

[1] Flemming H.-C., Wingender J., Szewzyk U., Steinberg P., Rice S.A., Kjelleberg S. (2016). Biofilms: An emergent form of bacterial life. Int. Rev. Microbiol..

[2] Hall-Stoodley L., Costerton J.W., Stoodley P. (2004). Bacterial biofilms: From the natural environment to infectious diseases. Nat. Rev. Microbiol..

[3] Petrova O.E., Sauer K. (2016). Escaping the biofilm in more than one way: Desorption, detachment or dispersion. Curr. Opin. Microbiol..

[4] Díaz-Pascual F., Hartmann R., Lempp M., Vidakovic L., Song B., Jeckel H., Thormann K.M., Yildiz F.H., Dunkel J., Drescher K. (2019). Breakdown of *Vibrio cholerae* biofilm architecture induced by antibiotics disrupts community barrier function. Nat. Microbiol..

